# 本科生综合实验:磁性固相萃取-高效液相色谱测定茶叶样品中的苯脲类除草剂

**DOI:** 10.3724/SP.J.1123.2024.04035

**Published:** 2024-11-08

**Authors:** Zhuang LIU, Ying SU, Dongwei YANG, Limei LI, Xu XU

**Affiliations:** 1.沈阳农业大学理学院, 辽宁 沈阳 110866; 1. College of Science, Shenyang Agricultural University, Shenyang 110866, China; 2.辽宁大学化学院, 辽宁 沈阳 110036; 2. College of Chemistry, Liaoning University, Shenyang 110036, China

**Keywords:** 综合化学实验, 磁性固相萃取, 高效液相色谱, 农药分析, 生物质材料, comprehensive chemical experiment, magnetic solid-phase extraction (MSPE), high performance liquid chromatography (HPLC), pesticide analysis, biomass materials

## Abstract

在教育部推进新农科建设和学科交叉融合的背景下,本研究设计了一项综合化学实验,旨在提升学生在生物质功能材料制备及农药残留检测中的实践技能。实验采用天然丝瓜络合成氮掺杂磁性多孔碳材料,应用于磁性固相萃取(MSPE)技术,结合高效液相色谱法(HPLC)检测茶叶样品中的苯脲类除草剂灭草隆。该实验流程包括材料合成、表征、MSPE条件优化、吸附性能研究及HPLC分析,体现了科学性和系统性,为学生提供了一个全面的学习平台。本实验不仅加深了学生对材料特性与应用之间关系的理解,而且提高了实验设计和问题解决能力。通过科学前沿、课程思政与实验教学的结合,不仅激发了学生的科研兴趣,培养了创新思维和实践能力,还加强了学生的社会责任感和历史使命感,实现了实验教学的全面育人目标,为培养具有全球视野和社会责任感的高素质人才奠定了基础。

## Abstract (en)

Against the backdrop of the Ministry of Education’s promotion of new agricultural science construction and interdisciplinary integration, a comprehensive chemistry experiment to enhance the practical skills of students in preparing biomass functional materials and detecting pesticide residues was designed. Natural loofah was utilized as a precursor in synthesizing nitrogen-doped magnetic porous carbon materials, which were then applied in a magnetic solid-phase extraction (MSPE) technique. Subsequently, high performance liquid chromatography (HPLC) was employed to analyze and detect the phenylurea herbicide monuron in tea. The experimental process included material synthesis, characterization, optimization of the MSPE conditions, adsorption performance studies, and HPLC, reflecting its scientific, systematic nature and providing a comprehensive learning platform for students. It not only deepens student understanding of the relationship between material characterization and application, but also improves their experimental design and problem-solving capacities. Moreover, by integrating cutting-edge science, ideological and political education, and experimental training, it not only stimulates student interest in scientific research and cultivates innovative thinking and practical skills, but also strengthens their feelings of social responsibility and historical mission. This approach realizes the comprehensive educational goals of experimental training and lays the foundation for nurturing high-quality talent with a global perspective and sense of social responsibility.

在新农科教育改革的推动下,我国农林高校正致力于构建跨学科教育模式,通过农学与工程、理科、医学、文科等学科的深度融合,培养具有创新精神和科研素养的复合型人才^[1]^。生物质可循环利用功能材料作为新材料产业的重要方向,利用农林生物质资源开发环境友好型材料,成为农工融合的关键点^[2]^。综合性化学实验课程作为高等教育的一部分,不仅巩固了学生的化学基础知识和实验技能,而且通过模拟实际问题,提升了学生的综合应用能力和团队合作精神。国内高校正将新材料、新技术整合入实验教学,以培养符合社会需求的创新型人才^[3⇓⇓⇓-7]^。本教学团队基于生物质材料和磁性固相萃取技术的研究^[8⇓⇓-11]^,将科研成果转化为教学资源,设计了综合性化学实验,旨在增强学生的实验技能,激发科研兴趣,提升创新思维和实践能力。

随着农业科学生产的发展,农药的广泛使用引发了环境和食品安全问题。苯脲类除草剂因其使用广泛、降解慢和有毒性而备受关注^[12]^。苯脲类除草剂对人体内分泌系统有害,具有潜在致癌作用,能在土壤中长期存在,污染水源和食物链^[13,14]^。因此,检测农产品和环境中的苯脲类除草剂残留至关重要。高效液相色谱法(HPLC)因具有高选择性、灵敏度和准确性,成为检测农药残留的主要方法^[15]^。然而,由于样品复杂和残留浓度低,检测前需采用预处理技术如磁性固相萃取(MSPE)富集待测组分,提高检测的准确性和灵敏度。MSPE技术结合磁性分离和固相萃取的优势,通过使用磁性材料吸附目标分析物,然后利用外部磁场分离,简化了样品预处理过程。MSPE技术操作简便、省时高效,且磁性吸附剂可功能化修饰并循环使用,已广泛应用于食品安全、环境监测和生物医药等领域^[16⇓-18]^。

本实验利用天然丝瓜络制备氮掺杂磁性多孔碳材料(N-MPC),旨在吸附和检测茶水中的苯脲类除草剂灭草隆(monuron)残留。实验流程包括材料合成与表征、MSPE条件优化、吸附性能评估和HPLC分析,融合了材料化学、分析化学、仪器分析和食品化学等学科知识。该实验设计强调了理论与实践的结合,体现了跨学科综合教学的特性,促使学生深入理解化学组分、材料性能及其在食品安全检测中的应用,并能提升学生的数据采集、处理和综合分析技能。

## 1 实验部分

### 1.1 仪器、试剂与材料

D5000 X-射线衍射仪(XRD,Siemens,德国), SU8000扫描电子显微镜(SEM,Hitachi,日本), LC-20A高效液相色谱仪,配备SPD-16紫外检测器(Shimadzu,日本), GSL-1700X管式炉(合肥科晶材料技术有限公司), Vortex-5旋涡混合器(其林贝尔仪器制造有限公司), KQ5200型超声波清洗器(昆山市超声仪器有限公司), HT190台式高速离心机(湖南湘仪实验室仪器开发有限公司), YRE-2012旋转蒸发器(巩义市予华仪器有限责任公司), LE104E/02分析天平(梅特勒-托利多仪器(上海)有限公司)。

尿素(CO(NH_2_)_2_)、九水硝酸铁(Fe(NO_3_)_3_·9H_2_O)、氢氧化钾购自国药控股化学试剂有限公司(沈阳)。灭草隆购自阿拉丁生化科技股份有限公司(上海),用乙腈配制质量浓度为1 mg/mL的标准溶液,保存在4 ℃的冰箱中,用超纯水稀释得到不同浓度的系列标准溶液。用Milli-Q水系统(Millipore, USA)获得超纯水。丝瓜络和茶叶样品购自当地市场(沈阳)。

### 1.2 色谱分析条件

C18色谱柱(150 mm×4.6 mm, 5.0 μm);流动相为乙腈-水(50∶50, v/v),流速为1.0 mL/min;检测波长为245 nm;进样量10 μL,柱温30 ℃。

### 1.3 实验步骤

#### 1.3.1 实际样品处理

取5 g茶叶于烧杯中,用100 mL沸腾的蒸馏水冲泡,用保鲜膜封好,静置10 min,不加(空白样品)或加入(加标样品)相应体积的一定浓度的标准溶液,4000 r/min离心10 min后,上清液经0.45 μm聚醚砜膜过滤,在4 ℃储存备用。

#### 1.3.2 N-MPC制备(教师制备,视频演示)

通过高温碳化方法制备N-MPC材料,过程如图1所示。首先,将干丝瓜络用超纯水洗涤5次,然后在60 ℃烘箱中烘干。取3 g干燥丝瓜络,剪成小片,放入三口烧瓶中。然后,分别称取12 g Fe(NO_3_)_3_·9H_2_O和36 g CO(NH_2_)_2_,分别溶解于60 mL乙醇和超纯水中,制得溶液A和B。将溶液A和B混合均匀,倒入含丝瓜络的烧瓶中,水浴加热至70 ℃保持3 h,取出丝瓜络,干燥12 h。将干燥后的丝瓜络与0.7 g氢氧化钾粉末混合均匀,放入磁舟中,在氮气氛围下,以5 ℃/min升温至1000 ℃煅烧1 h。最后,将煅烧后的材料研磨成粉末,得到N-MPC吸附剂,密封备用。

**图1:**
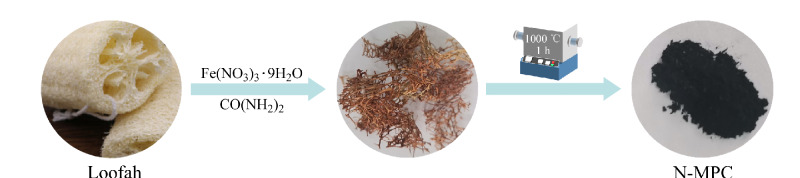
N-MPC合成示意图

该制备过程由实验教师先行完成,将录制完整的合成过程教学演示视频作为先导课程,辅助学生学习和巩固材料合成与表征方面的知识。

#### 1.3.3 MSPE过程

N-MPC的MSPE过程如图2所示。用分析天平准确称取5 mg N-MPC吸附剂并分散于5 mL样品溶液中,然后采用小型旋涡混合器以1000 r/min的转速剧烈振摇5 min,混匀后用高强磁铁收集吸附剂,再将其加入到3 mL甲醇溶液中,在超声条件下进行洗脱,1 min后再利用磁铁将吸附剂与洗脱液分离。得到的洗脱液旋转蒸发干燥后,用100 μL乙腈复溶。最后,用0.22 μm聚四氟乙烯膜过滤洗脱液,用HPLC-UV分析。

**图2:**
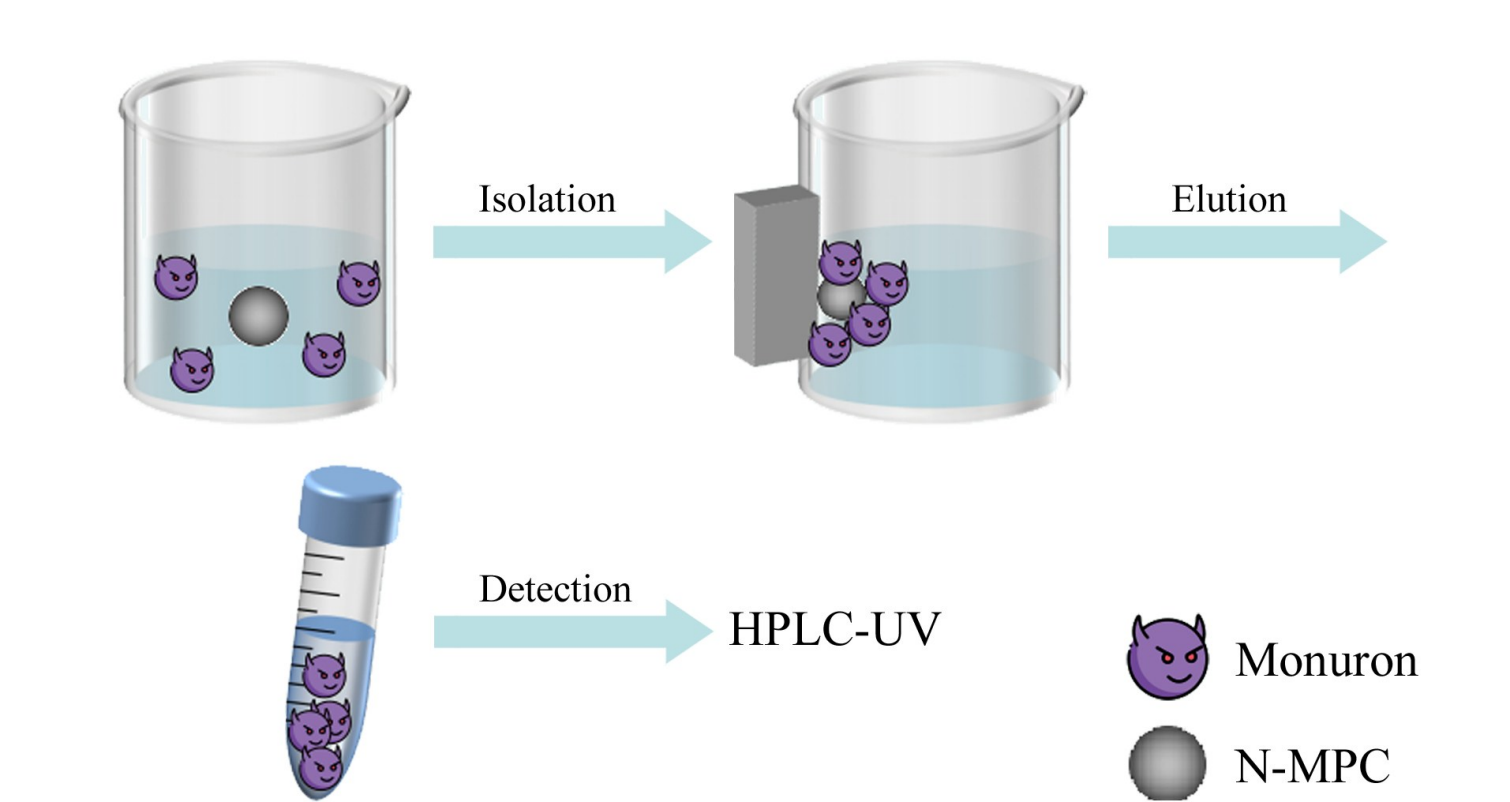
MSPE流程图

为使N-MPC材料达到最优的萃取效果,本实验选用质量浓度为10 μg/L的灭草隆标准溶液,采用单因素控制变量法,对影响吸附实验的吸附剂质量、吸附时间、样品溶液的pH和离子强度等因素进行全面分析和优化。

## 2 结果与讨论

### 2.1 材料表征

采用SEM对丝瓜络及N-MPC的微观形貌进行分析。将制备的N-MPC用适量的乙醇分散在小试管中,并通过超声处理使分散更均匀。然后,将分散好的样品滴在硅片上,让其自然晾干。使用导电胶带固定硅片,确保样品在SEM样品台上稳固固定,避免在高真空环境下由于磁场作用而移动或旋转。图3a是用去离子水洗净干燥的丝瓜络,可以看到丝瓜络表面自然状态下呈现较多的褶皱。图3b是制备得到的N-MPC材料,丝瓜络表面含有大量纳米颗粒,证明了Fe和Fe_3_C均匀分布在表面。

**图3:**
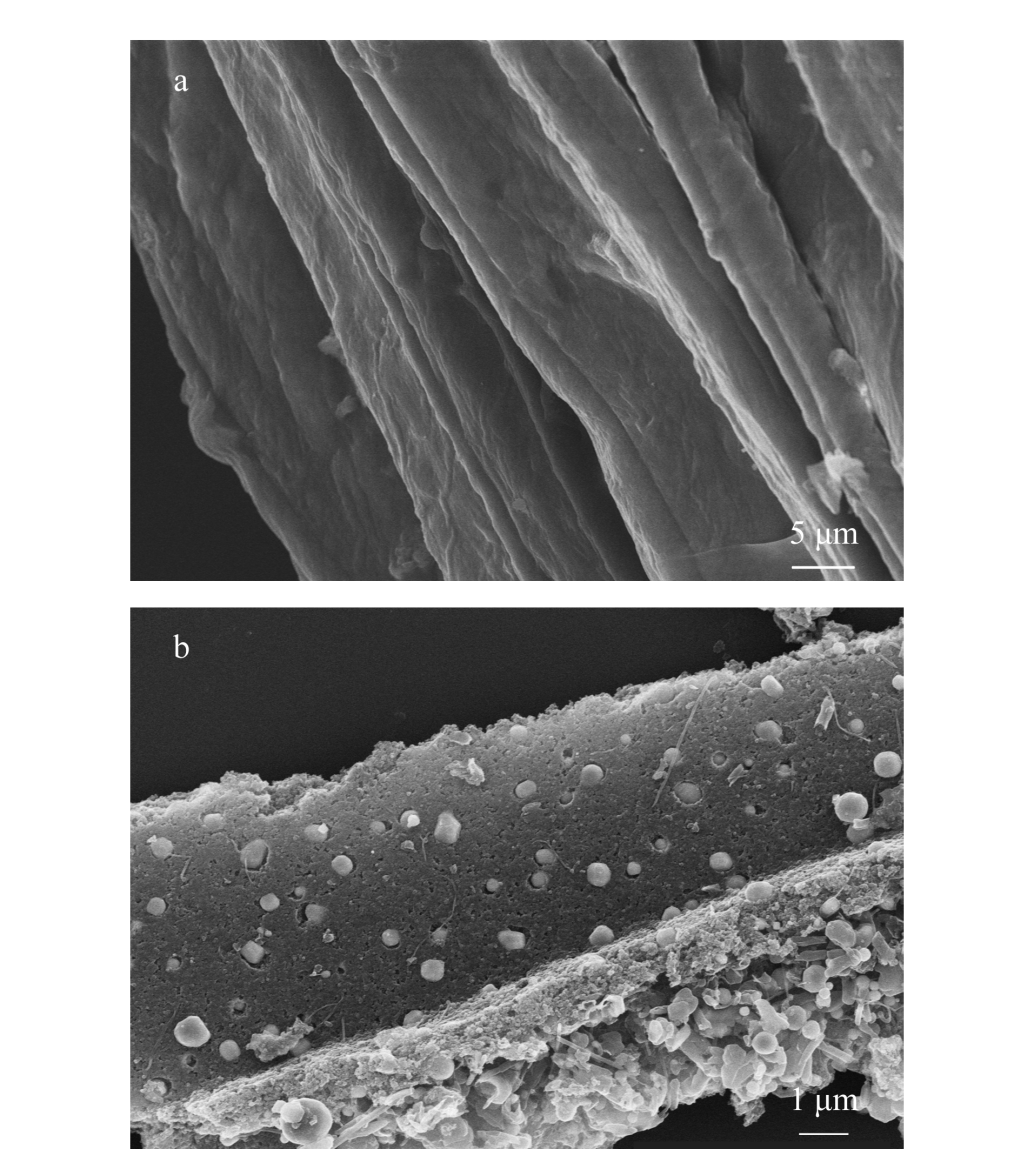
(a)丝瓜络及(b)制备的N-MPC的SEM图谱

采用XRD对N-MPC的晶体结构进行表征。图4展示了N-MPC的XRD图谱,其中在44.7°、65.0°和82.3°处观察到的特征衍射峰分别对应于Fe(JCPDS: 87-0721)的(110)、(200)和(211)晶面。衍射峰(43.2°、50.2°、73.8°)对应为Fe_3_C(JCPDS: 63-0040)。此外,在26.5°处观察到的宽衍射峰与碳材料中堆叠的(002)晶面相关联,该宽峰的存在进一步证实了N-MPC材料的成功合成。

**图4:**
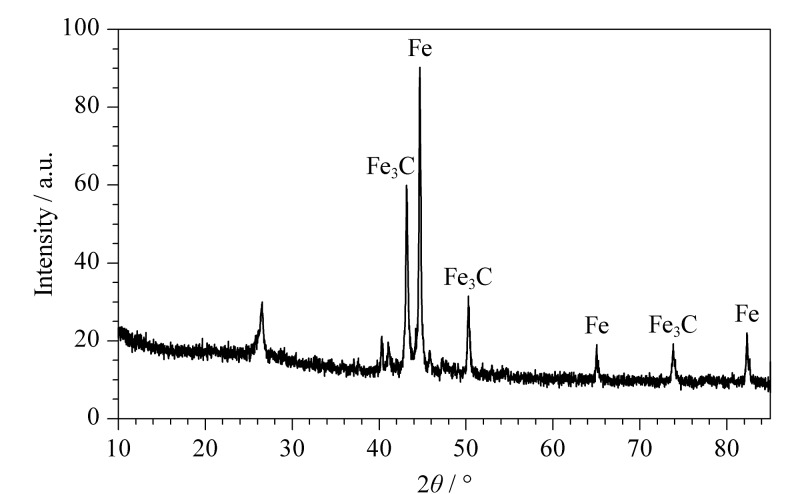
N-MPC的XRD图谱

### 2.2 MSPE实验条件考察

在实验中,N-MPC的用量对灭草隆的吸附效率具有显著影响。不足的N-MPC可能导致吸附不完全,而过量则增加分析成本和时间。如图5a所示,随着N-MPC用量增加,灭草隆回收率逐渐提升,并在5 mg时达到峰值,因此确定5 mg为最佳吸附剂量。此外,考察了不同吸附时间(1~20 min)对回收率的影响。如图5b所示,从1 min至5 min,回收率随时间增加而提高,而5 min至20 min,回收率趋于稳定,表明吸附已达到平衡,故选择5 min作为吸附时间。

**图5:**
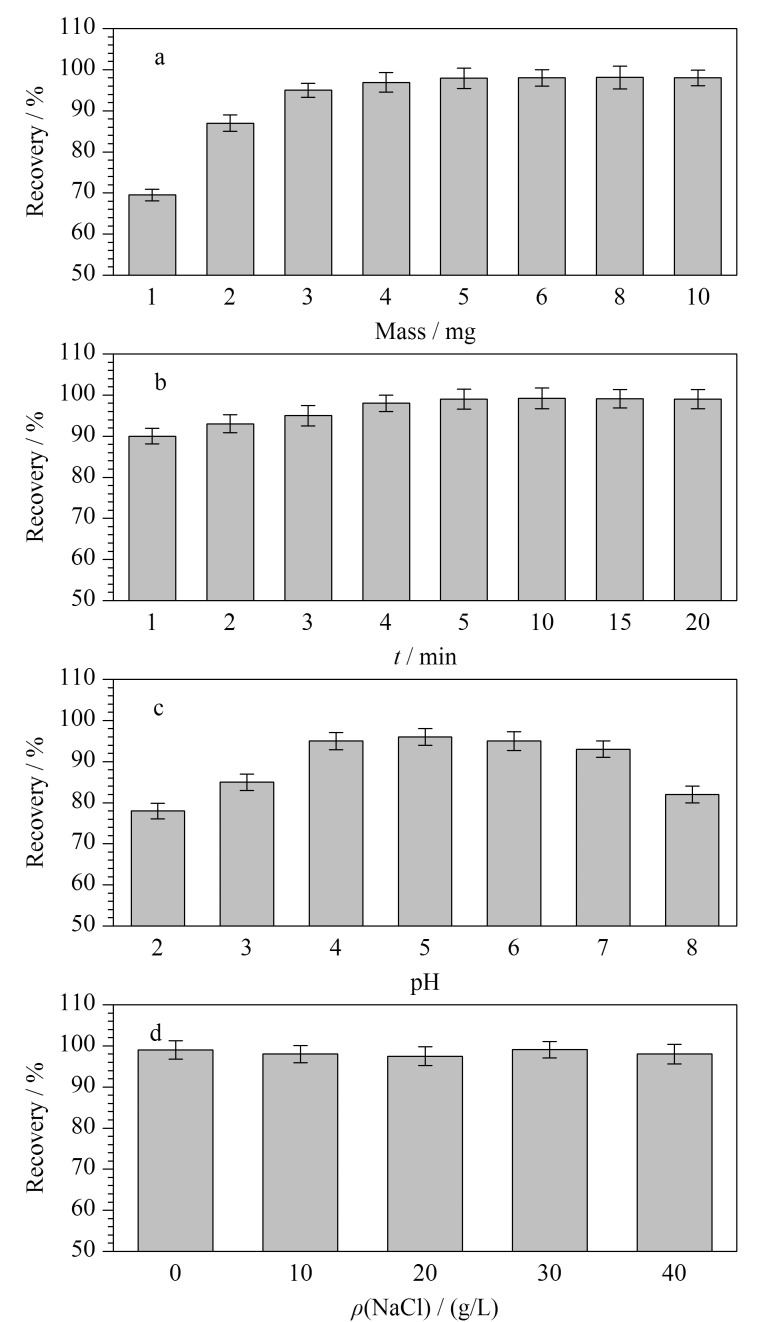
(a)N-MPC的质量、(b)吸附时间和(c)样品溶液的pH和(d)盐质量浓度对吸附性能的影响(*n*=3)

样品溶液的pH值对吸附剂表面的电荷状态及分析物存在形式有重要影响。实验发现(图5c),当pH值为4~7时,灭草隆的回收率较高,而当pH值超过7时回收率降低,这可能是因为灭草隆在强酸碱条件下容易发生分解。鉴于实验所用水的pH值为6.3,因此无需额外调节pH。

离子强度的增加通常可提高萃取效率,但过高的离子强度会增加溶液黏度,影响扩散吸附。实验通过向样品溶液中添加不同量的氯化钠(0~40 g/L)来研究离子强度对灭草隆吸附回收率的影响。从图5d中可以看出,随着氯化钠质量浓度的增加,灭草隆的回收率略有下降,因此在后续实验中不添加氯化钠。

在优化洗脱条件的研究中,选取合适的洗脱溶剂对提高洗脱效率至关重要。本研究对比了甲醇、乙醇、乙腈和丙酮4种有机溶剂的洗脱能力,其中甲醇的洗脱效率(98.5%)最高。进一步探究了甲醇用量(1~4 mL)对洗脱效果的影响,发现3 mL甲醇用量能够达到最佳洗脱效率(99.0%)。此外,洗脱时间的长短也是影响洗脱效率的关键因素。实验优化了不同洗脱时间(0.5~5 min)对洗脱效率的影响,洗脱时间为1 min时洗脱效率为98.9%,已足够实现目标分析物的洗脱。因此,选择3 mL甲醇作为洗脱溶剂,洗脱时间为1 min。

### 2.3 吸附性能研究

在室温条件下,通过测试N-MPC的吸附等温线评估其对灭草隆的吸附能力。将5 mg吸附剂加入到20 mL不同质量质量浓度(30~400 mg/L)的标准溶液中,利用旋涡混合器振荡5 min。实验数据采用Langmuir和Freundlich等温线模型(见式(1)与式(2))进行分析。


(1) $$\frac{C_{e}}{q_{e}}=\frac{1}{K_{L}q_{m}}+\frac{C_{e}}{q_{m}}$$

(2) $$\ln q_{e}=\ln K_{F}+\frac{1}{n}\ln C_{e}$$

其中,平衡吸附容量和最大吸附容量分别用*q*_e_(mg/g)和*q*_m_(mg/g)表示。平衡质量质量浓度为*C*_e_(mg/L), Langmuir和Freundlich常数分别为*K*_L_(L/mg)和*K*_F_((mg/g)/(mg/L)^1/^*^n^*)。两种模型的拟合线性如图6a~c所示,Langmuir模型具有较高的相关系数(*R*^2^=0.9936),说明该过程更符合Langmuir模型,灭草隆在N-MPC上的吸附过程属于单分子吸附。N-MPC对灭草隆的最大吸附容量分别为89.36 mg/g。学生通过测定材料的等温吸附曲线得出材料的饱和吸附量,该参数是评价吸附剂的重要指标之一,该实验过程能让学生充分体会到实验可以为工业化提供必要的生产参数,鼓励学生夯实专业知识素养。

**图6:**
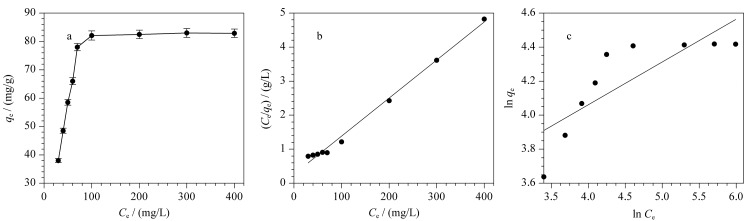
(a)灭草隆的平衡吸附容量与平衡浓度的关系(*n*=3),(b)Langmuir模型和(c)Freundlich模型拟合的平衡吸附等温线

### 2.4 方法学考察

在最佳实验条件下,通过测定不同加标水平(2、5、10、25、50、100、200 μg/L)的标准溶液获得方法的标准曲线。根据线性范围、线性相关系数(*R*^2^)、检出限(LOD,*S/N*=3)、定量限(LOQ,*S/N*=10)对分析方法进行了评价。灭草隆的线性范围为2~200 μg/L,相关系数为0.9987, LOQ和LOD的分别为0.46 μg/L和1.53 μg/L,表明该方法能够检测痕量的灭草隆。为了评估方法的重复性,对标准溶液进行3次平行测定,得到的相对标准偏差(RSD)为3.0%,这表明该方法具有良好的重复性。富集倍数(EF)是衡量分析方法效率的一个重要参数,其计算公式如式(3)所示:


(3) EF=*C*_1_/*C*_0_

其中,*C*_1_(μg/L)为洗脱溶剂中灭草隆的最终质量浓度,*C*_0_(μg/L)为样品溶液中灭草隆的初始质量浓度。本实验中灭草隆的EF为46,这表明该方法能够有效富集并检测痕量灭草隆。

### 2.5 实际样品检测

为了考察该方法的适用性,对3种不同茶叶样品进行分析,结果表明,样品中未检测到灭草隆,在灭草隆的出峰位置也没有明显的干扰峰。对样品进行了不同水平的加标回收试验,如表1所示,茶叶样品中灭草隆的回收率为87.5%~96.0%(RSD=1.9%~3.5%),表明该吸附剂具有较高的回收率和良好的精密度,可用于实际样品中微量灭草隆的富集和检测。其中一个茶叶样品加入灭草隆标准品(2.0 μg/g)前后的典型色谱图见图7。

**表1:** 3种实际样品中灭草隆在0.2、0.4、2.0 μg/g 3个水平下的加标回收率(*n*=5)

Spiked/(μg/g)		Sample 1			Sample 2				Sample 3		
		Recovery/%	RSD/% (*n*=5)		Recovery/%	RSD/% (*n*=5)			Recovery/%	RSD/% (*n*=5)	
0.2	92.3		1.9		87.5		2.5		92.8		2.7	
0.4	94.5		2.1		93.3		2.6		93.3		3.3	
2.0	96.0		3.0		90.8		3.5		95.4		2.9	

**图7:**
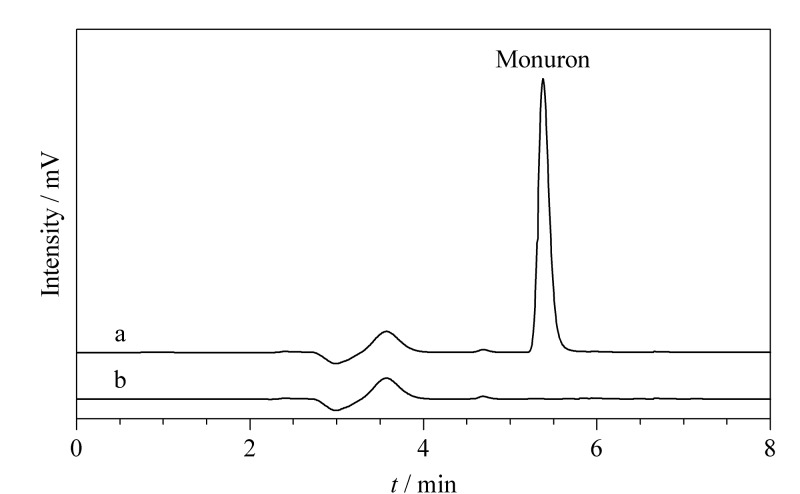
茶叶(a)加标(2.0 μg/g)样品及(b)空白样品中灭草隆的色谱图

## 3 教学实践与反思

### 3.1 实验安排

本实验采用线上线下混合式的教学方法,实验教学中尽管材料表征测试、吸附性能测试等环节各自独立,但学生间的交流与合作对于培养他们的综合性实验技能至关重要,可采取课前准备、团队分工、跨组交流、共同讨论及教师引导等措施,具体实施过程如下。

课前:学生课前通过线上平台预习相关文献、观看演示视频、学习软件操作和仪器分析,提前了解实验的各个环节和要求,以提升独立思考能力。学生需制定实验方案,与教师讨论并优化方案,旨在激发学生的创新思维,培养对环保农药的认知和职业道德。

课中:实验建议为10学时,按学生人数分为4个小组,每个小组负责不同的任务模块。通过小组讨论和计划,学生可以明确各自的职责和任务。具体分工如下:(1)样品制备与分析。第一组同学负责配制不同质量浓度的标准溶液,绘制标准曲线,并通过液相色谱检测优化萃取条件;(2)材料表征。第二组同学利用SEM和XRD对吸附剂进行表征分析;(3)吸附性能研究。第三组同学研究吸附剂的性能,实验内容可根据需要增加吸附动力学研究,额外增加2学时;(4)定量分析。第四组同学通过方法标准曲线对实际样品进行定量分析。采用任务驱动式教学方法,教师安排小组间交流会议,让各组分享他们的发现和进展,促进信息共享和知识交流。通过小组合作,培养学生的沟通、团队协作能力,以及“物质-结构-性能”的科研思维,提升分析和解决问题的创新能力。同时,强调实验记录的实事求是和科学严谨性。

课后:实验结束后,鼓励学生参与深入讨论,提出改进建议,并完成实验报告。这个过程有助于学生整合不同模块的知识,形成系统的理解。教师在教学过程中应发挥引导和反馈作用,帮助学生理解不同模块之间的联系,以及如何将这些知识应用到实际问题的解决中,形成闭环教学过程。通过数据处理、论文写作和过程反思有助于培养学生的科学思维方法,提高学生的综合能力,同时使学生了解新型农药残留分析的重要性,激发学习兴趣,培养爱国情怀,鼓励科学创新。

### 3.2 教学反馈

在完成一个教学周期(2023秋)后,教师对学生进行了调研,以了解学生对实验的评价和收获。调研结果显示,学生对这一创新性实验表现出了极大的兴趣和积极反响。学生普遍认为,涉及生物质衍生碳材料的合成及其在萃取和吸附性能研究中的应用,不仅具有趣味性和创新性,而且通过前沿文献的调研,他们的视野得到了显著拓展,学生们初步构建了从“资料查阅、材料制备、表征测试到分析应用”的科研逻辑思维框架。他们在自主学习文献、解决实验问题的过程中,自主学习能力和创新实践能力均有所增强。此外,学生对前沿科学研究的认识更加深入,对科学探索的兴趣亦随之提升。

综上,实验达到了预期的教学效果,不仅增强了学生的专业知识和技能,也激发了他们对科学研究的热情和追求。

### 3.3 教学反思与改进

为进一步提高教学质量,计划采取以下改进措施。(1)SEM操作优化:鉴于磁性材料可能对SEM造成干扰,建议采取磁性屏蔽、样品处理(如涂层或固定)、专业操作培训及设备定期维护等措施,以确保实验的安全性和有效性。同时,硅片或其他载体需要保持清洁,以避免样品污染。分散后的样品应均匀覆盖在硅片上,避免过度集中或分散不均。(2)生物质材料应用多样化:本实验成功将丝瓜络转化为有价值的碳材料,展示了农业废弃物再利用的潜力。未来可探索银杏叶、柳絮、咖啡渣等其他生物质材料,以拓展吸附材料或生物炭在环境监测中的应用。(3)HPLC技术教学扩展:目前实验仅限于分析一种苯脲类除草剂。为深化学生对HPLC在多组分分析中的理解,建议增加3~5种不同类别的农药,以全面学习其定性与定量分析技术。(4)实验安全性强化:针对高温管式炉操作的安全考量,建议采用视频演示、虚拟仿真及水浴或微波辅助合成等替代方法,使学生在安全环境下掌握生物炭制备的关键技术。这些改进措施将提高实验教学的安全性和效果,深化科研实践,同时培养学生的创新能力和解决实际问题的技能。

## 4 结论

在师生的共同努力下,实验课程经过深入探讨和优化,并引入了SEM和XRD表征技术。样品前处理和液相色谱法的结合不仅丰富了课程内容,也提升了学生的兴趣和参与度。学生对分析仪器有了更深刻的理解,体验了完整的分析流程。教师通过观察学生操作,及时调整教学方法,有效提高了教学质量和自身技能。

实验不仅在实验教学层面取得进展,更在思政教育方面发挥了重要作用。绿色化学理念和环保农药的探索,增强了学生对化学技术在可持续发展中作用的认识,培育了他们的社会责任感和环境伦理观。问题解决和创新思维的训练激发了学生的爱国热情和创新精神,为培养具有国际视野和创新能力的新时代青年奠定了基础。教师在教学中融入思政元素,使学生在掌握科学技术的同时,理解并承担起建设科技强国的责任。通过科学前沿、课程思政与实验教学的融合,提升了学生的实验技能,强化了其历史使命感,实现了全面育人的教育理念,为培育具有全球视野和社会责任感的高素质人才提供了重要支撑。
